# The Ac/N-Degron Domain of MARCHF6 E3 Ubiquitin Ligase and Its Role in Regulating Ferroptosis

**DOI:** 10.3390/cells14130954

**Published:** 2025-06-22

**Authors:** Hope Omoniyi, Grace Hohman, Mohamed Eldeeb

**Affiliations:** Department of Chemistry, Illinois State University, Normal, IL 61790, USA; hoomoni@ilstu.edu (H.O.); gehohma@ilstu.edu (G.H.)

**Keywords:** protein degradation, proteolysis, proteasome, N-terminal acetylation, ferroptosis, ubiquitin

## Abstract

Ferroptosis is a form of cell death characterized by iron and reactive oxygen species accumulation. Notably, this mode of cell death has been shown to exhibit significant implications for aging-related disorders including tumorigenesis and neurodegeneration. Nonetheless, the intricate underlying molecular mechanisms of ferroptosis and their differential roles in the molecular etiology of these diseases are still elusive. Elucidating the precise molecular mechanisms underlying ferroptosis is, thus, important for understanding the molecular basis of these diseases and unveiling potential therapeutic targets. MARCHF6 is an E3 ub ligase involved in regulating various cellular processes throughout the cell including ferroptosis. Research findings by Yang et al. identified a novel role of MARCHF6 E3 ub ligase in recognizing Ac/N-degron bearing substrates, which includes pro-ferroptotic and anti-ferroptotic proteins, demonstrating a regulatory role for MARCHF6 in fine-tuning ferroptosis. Herein, we highlight these recent findings and discuss the potential role of MARCHF6 in modulating ferroptosis pointing to the emerging role of MARCHF6 as a potential therapeutic target for treating ferroptosis-related diseases.

## 1. Introduction

While apoptosis and necrosis are among the most well-known forms of cell death [1], research conducted by Dixon et al. in 2012 identified a non-apoptotic form of cell death called ferroptosis [2]. This form of cell death is characterized by unrestrained lipid peroxidation, a chemical reaction between reactive oxygen species (ROS) and polyunsaturated fatty acids. In the cell, this can be detrimental to the plasma membrane—which consists of fatty acids—and has been shown to lead to its rupture. The biochemical processes that occur to initiate ferroptosis are largely spontaneous and uncatalyzed due to imbalances in iron and redox homeostasis [3]. Ferroptosis does not have the same characteristics as apoptosis, like cell shrinkage and apoptotic body formation, or features of necrosis like inflammation. The morphological manifestations of ferroptosis are unique and include mitochondrial shrinkage and a reduction in mitochondrial cristae [4]. Ferroptosis is present in cancers and neurodegenerative diseases, making this mechanism an important area for research [5,6]. Recently, Yang et al. elucidated key aspects of this pathway involving the protein MARCHF6 [7]. In this review we aim to discuss the key findings from this study, highlighting the important role of MARCHF6 in regulating ferroptosis.

**Figure 1:**
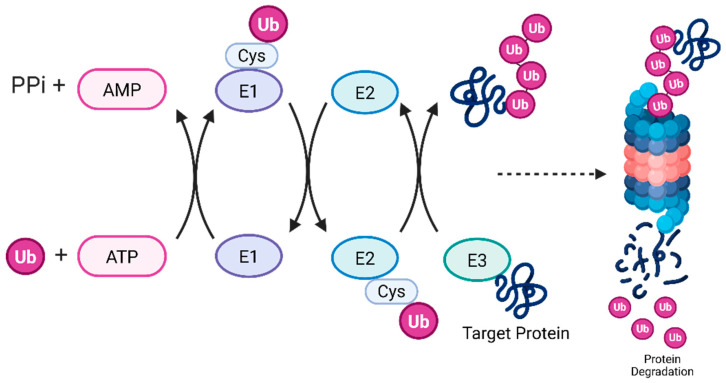
A schematic depicting the enzymatic cascade involved in the ubiquitin–proteasome system. The first step involves E1 utilizing ATP to activate ubiquitin. Next, ubiquitin is shuffled from E1 to E2. Finally, E3 marks the target protein with ubiquitin, marking it for degradation via the proteasome. This figure was crafted with the help of BioRender (https://app.biorender.com/, accessed on 3 February 2025).

**Figure 2:**
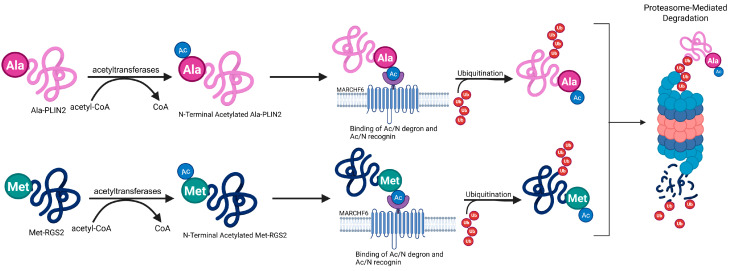
A schematic depicting N-terminal acetylation of Ala-PLIN2 and Met-RGS2. This N-terminal acetylation generates an Ac/N-degron, and these proteins are recognized by MARCHF6, which mediates their ubiquitination and their subsequent proteasomal degradation. This figure was crafted with the help of BioRender.

MARCHF6 E3 ubiquitin (ub) ligase (MARCHF6) is a large protein embedded in the endoplasmic reticulum of eukaryotic cells with a molecular mass of 102,545 Da [8]. It is the most common Ac/N recognin in mammals and functions within the Ac/N-degron pathway to recognize N-terminal acetyl groups of proteins for proteasomal degradation [9]. E3 ubiquitin ligases work as part of the ubiquitin–proteasome system (UPS) that works with the ub-activating E1 enzymes and the ub-conjugating E2 ligase enzymes to ubiquitinate proteins and mark them for degradation (Figure 1) [10,11]. Mechanistically, the ubiquitination cascade is initiated by ub attaching to E1 ub-activating enzymes, a transfer that is ATP-dependent and forms a thioester bond. Ubiquitin is then transported to the cysteine residue of an E2 ub-conjugating enzyme. Lastly, an E3 ub-ligase enzyme specifically recognizes the target protein and facilitates the transfer of ub. This leads to polyubiquitination and is a signal to the cell for protein degradation and various cellular processes [12]. The Ac/N degron pathway has implications in various processes throughout the cell including protein quality, circadian rhythm, and the stress response [13,14]. Additionally, MARCHF6 can regulate ferroptosis through the Ac/N degron pathway by recognizing the N-terminal acetylated group as a signal for degradation called an Ac/N-degron. The proteins that exhibit N-terminal acetylated degrons are a signal to the cell that can mark the protein for ubiquitination, which may ultimately lead to proteolysis (Figure 2). Ac/N degrons can be found on proteins like the regulator of G-protein signaling RGS2 [15] and a key regulator of lipid homeostasis, PLIN2 [16]. MARCHF6’s Ac/N recognition domain preferentially binds to these N-terminal acetylated proteins and mediates their degradation. Inhibition of MARCHF6’s ability to recognize Ac/N degrons through the Ac/N degron pathway prevents the proteolysis of RGS2 and PLIN2 and resists ferroptosis. However, the precise mechanism for the recognition of Ac/N degrons by the Ac/N degron domain remains unknown. To shed light on this pathway, the authors performed methodical assessments of the outer cytosolic-facing regions of MARCHF6, utilizing several biochemical techniques to define the complete Ac/N domain of MARCHF6 in human cells and Doa10, its counterpart in yeast cells.

## 2. How Does MARCHF6 Interact with Ac/N Degrons to Facilitate Degradation?

MARCHF6 recognizes the N-terminal acetylated portion of proteins and marks them for degradation, which occurs via the Ac/N degron pathway. To understand the connection between ferroptosis and MARCHF6, the delineation of MARCHF6 and its Ac/N domain is necessary. MARCHF6 must act to recognize the appropriate proteins for degradation by recognizing the N-terminal acetylated region of Ac/N degrons. However, MARCHF6 must also be able to recognize non-Nt acetylated proteins through alternative sites and spare them from degradation. Researchers posited that both N-terminal acetylated and non-Nt acetylated proteins play a role in this MARCHF6 recognition of Ac/N degrons. In turn, MARCHF6 plays a rheostatic role in the regulation of ferroptosis through the Ac/N degron pathway.

MARCHF6 is a vast transmembrane protein containing 14 membrane-spanning helices, 8 cytosolic-facing stretches, and a RING domain, and is regulated by the C-terminal region. The cytosolic regions of MARCHF6 are labeled Cn, with n indicating the series of the cytosolic region from the N-terminus [17]. Since Nt-acetylation ocurrs in the cytosol, the authors have suggested that one of the eight cytosolic regions of MARCHF6 is involved in the identification of Ac/N-degrons. In order to confirm this conjecture, the MARCHF6 of humans and mice and Doa10 of yeast evolutionary conserved residues were mutated into alanines. The authors created 25 discrete C-terminal tagged MARCHF6 mutants through chemical cross-linking and reciprocal co-immunoprecipitation–immunoblotting using HeLa cells. The C5-18 and the C5-19 region mutants did not co-immunoprecipitate with N-terminal Met-RGS2 (Nt-M-RGS2), indicating there were no protein–protein interactions. Conversely, the other alanine stretch mutants and the wild-type co-immunoprecipitated with M-RGS2, pointing to protein–protein interactions. In HeLa cells, similar results were seen with the natural C5-17 and C5-20 stretches co-immunoprecipitating with M-RGS2 or N-terminal Ala-PLIN2 (Nt-A-PLIN2), whereas mutated C5-18 and C5-19 did not.

Squalene monooxygenase (SM) is another substrate of MARCHF6 [18,19] that may bind to the Nt-transmembrane helices and central cavity rather than the C5 region. To ensure that SM does not bind to the C5 region, the authors replaced the wild-type start of the substrate, Nt-Met, with Nt-Pro. This alteration should inhibit the ability of MARCHF6 to recognize SM through the C5-18 and C5-19 regions if SM is recognized through the Ac/N terminal degradation pathway. Both the wild-type and mutated SM interacted with MARCHF6, indicating that the recognition of SM must be through another pathway—either in a cytosolic-facing region or a transmembrane domain. This leaves MARCHF6’s C5 region responsible for the recognition of Ac/N degrons.

## 3. The C5 Region of MARCHF6 Is Essential for Recognizing Ac/N Degrons

Further characterization of the Ac/N degron recognition pathways was conducted using a split-ub technique, where a transcription factor, LexA-VP16, was linked to C-terminal truncated MARCHF6. These various segments are described by indicating the length of the segment as a subscript, where the first residue was described until the end of the C-terminally truncated MARCHF6 fragment. The full-length, wild-type MARCHF6 containing residue 1-910, the C-terminal fragment containing residues 1-810, and the fragment containing amino acids 1-721 all interacted with Nt-M-RGS2 and Nt-A-PLIN2. The shorter segments, residues between 1 and 565, did not interact with Nt-M-RGS2 and Nt-A-PLIN2. The specific region of MARCHF6 that controls the Ac/N degron pathways and permits the binding and recognition of Ac/N degrons must lie after the 565 region due to the inability of prior residues to recognize Ac/N degrons. This leaves the C5 recognin to be located between MARCHF6^541-632^. Another split-ub technique was used to determine whether the C5 region of MARCHF6 is the sole region used as a recognition site for Ac/N degrons. To achieve this, the other cytosolic regions were tethered to the endoplasmic reticulum. The C5 region of MARCHF6 did not interact with P-PLIN2—a non-Nt-acetylatable substrate—but did interact with the Nt-acetylatable version of the same protein. Similarly, in HeLa cells, MARCHF6 541-632 only interacted amid Nt-acetylatable M-RGS2 and A-PLIN2. To understand the minimum region of C5 that plays a role in Ac/N degron recognition, the split-ub technique was used to further truncate the region into three parts. When it was made to interact with Nt-acetylatable A-PLIN2, only MARCHF6^552-579^ interacted. These findings suggest that this region is likely the most important for identifying Ac/N degrons.

The MARCHF6 Ac/N domain is evolutionarily conserved and programmed to recognize Ac/N degrons [7]. When conserved residues were mutated to express alanine instead of their native amino acid, recognition of Nt-acetylatable M-RGS2 and A-PLIN2 ceased. However, other MARCHF6 substrates like SM, ACSL4, and p53 were still able to bind with the alanine-expressing Ac/N domain. These findings suggest that these other ferroptotic factors must use a different binding site from Nt-M-RGS2 and Nt-A-PLIN2. Additionally, when the mutated alanine expressing Ac/N domain was exposed to Nt-M-RGS2 and Nt-A-PLIN2, degradation did not occur. Conversely, the wild-type MARCHF6 Ac/N domain was able to recognize these substrates and degrade them. Thus, the native Ac/N domain must be specifically evolved to recognize these substrates. Changes that occur to this domain will inhibit the degradation of Nt-M-RGS2 and Nt-A-PLIN2.

## 4. MARCHF6 Regulates Ferroptosis Through the Ac/N Degron Pathway

The connection linking the Ac/N degron pathway of MARCHF6 and ferroptosis was measured by the extent of lipid reactive oxygen species (ROS) in the cell. Lipid ROS can attack fatty acids, like the phospholipids of the cell membrane, and destroy them via lipid peroxidation [6,20]. This process is a key component of ferroptosis. Interestingly, overexpression of Nt-M-RGS2 and Nt-A-PLIN2 in HeLa and A549 cells reduced the levels of lipid ROS and increased the vitality of the cell. Non-Nt-acetylatable substrates similar to Nt-P-RGS2 and Nt-P-PLIN2 also greatly decreased the amount of lipid ROS found in the cell. This could be because MARCHF6 could not degrade the substrate fast enough, which is seen in the case where MARCHF6 was not able to degrade Nt-M-RGS2 and Nt-A-PLIN2 when they were overexpressed. Thus, PLIN2 can act as an inhibitor of ferroptosis both by promoting the formation of lipid droplets (LDs) and blocking against lipid peroxidation. Unfortunately, in other forms of cancer, PLIN2 and LDs can act to increase ferroptosis.

Given that PLIN2—a substrate of MARCHF6—can decrease ferroptosis, the authors explored the contribution of the Ac/N degron pathway of MARCHF6 in ferroptosis. Wild-type MARCHF6 recognized RGS2 and PLIN2 and signaled them for degradation via the Ac/N degron pathway. This resulted in lower levels of the substrates. However, the total amount of lipid ROS in the cell decreased, leading to viability of the cancer cell. These results agree with previous research demonstrating that MARCHF6 suppresses ferroptosis [21]. Thus, the inhibition of MARCHF6 can halt the degradation of RGS2 and PLIN2 and could be used to enhance resistance to ferroptosis.

## 5. The Multifaceted Role of MARCHF6 in Regulating Ferroptosis

**Figure 3:**
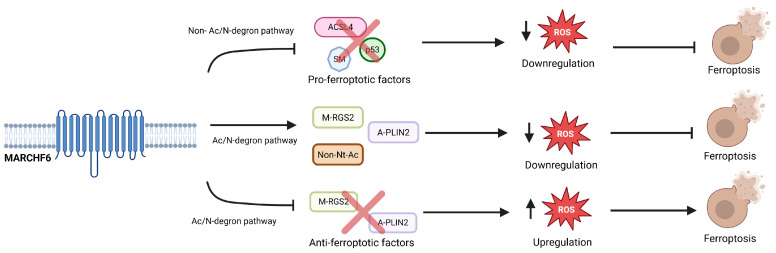
A schematic depicting the rheostatic role of MARCHF6 E3 ub ligase in ferroptosis by targeting pro-ferroptotic substrates and anti-ferroptotic substrates. MARCHF6 can counteract ferroptosis signaling by inhibiting pro-ferroptotic effectors causing a decrease in lipid ROS levels and resisting ferroptosis. Additionally, MARCHF6 can trigger ferroptosis signaling by degrading anti-ferroptotic factors and increasing lipid ROS levels. This figure was crafted with the help of BioRender.

MARCHF6 has been shown to suppress ferroptosis by facilitating the degradation of specific substrates. Understanding the mechanism of degradation and recognition is integral to understanding ferroptosis. The C5 region of MARCHF6 has been identified as the site of the Ac/N domain, the specific binding site lying between the C5 552 and 579 region. This region identifies Nt-acetylatable substrates like RGS2 and PLIN2 and marks the proteins for ub-mediated degradation. This system has now been demonstrated to regulate ferroptosis as well. MARCHF6 can regulate ferroptosis through the inhibition of pro-ferroptotic effectors causing the upregulation of anti-ferroptotic factors that were experimentally discovered through a mutant MARCHF6. However, MARCHF6 can also assist ferroptosis by degrading the substrates of the Ac/N degron pathway like N-terminal acetylated M-RGS2 and A-PLIN2 that inhibit ferroptosis. The dual function of MARCHF6 confers the rheostatic role of the MARCHF6 E3 ub-ligase in ferroptosis as it can affect pro-ferroptotic substrates and anti-ferroptotic substrates (Figure 3). However, these functions must exist in a delicate balance as cellular stress can alter the functions of MARCHF6.

## 6. Conclusions and Future Directions

Understanding the intricate role of MARCHF6 and its various substrates can provide further insights into understanding the molecular basis of regulation of ferroptosis in the context of cancer cell biology and neurodegeneration. The authors of this study provided new insights into the molecular mechanism of regulation of ferroptosis by delineating the recognition domain of MARCHF6 E3 ub ligase of the Ac/N degron pathway. Elucidating the full repertoire of the physiological substrates involved in this pathway could provide additional insights into the intricate mechanisms of ferroptosis. Further investigation of the role of the calcium cation in the induction of ferroptosis could expand our understanding regarding the various physiological triggers ferroptosis in various cells [22]. Furthermore, the location of MARCHF6 may play a role in its function. Identifying similar proteins in other organelles of the cell could lead to the discovery of proteins that have similar rheostatic modulation roles.

As knowledge of ferroptosis increases, modulating the process may be of interest to researchers for developing novel treatments. Inhibiting ferroptosis in cells associated with neurodegenerative diseases and various cancers may be key to halting and treating these conditions.

## Data Availability

Not applicable.
